# Acute Tension-Type Headaches Are Associated with Impaired Cognitive Function and More Negative Mood

**DOI:** 10.3389/fneur.2016.00042

**Published:** 2016-03-29

**Authors:** Andrew Paul Smith

**Affiliations:** ^1^Centre for Occupational and Health Psychology, School of Psychology, Cardiff University, Cardiff, UK

**Keywords:** tension-type headache, mood, memory, attention, psychomotor speed

## Abstract

**Background/aims:**

Research has shown that migraine is often associated with memory problems. There have, however, been few studies of tension-type headache (TTH) and cognition. People who report frequent headaches often report high levels of negative affect. However, less is known about the acute effects of TTH on mood. To address these gaps in our knowledge, two studies examined the effects of acute TTH on ­cognitive performance and mood.

**Methods:**

Both studies involved one group of participants completing a battery of tasks when they had a TTH and when they had no headache. Another group (the control) was headache free on both occasions. Duration of the headache was >30 min and <4 h. In the “no headache” condition, the participants were headache free for at least 24 h. In the first study, 12 participants (6 with TTH and 6 controls) completed a computerized battery measuring mood and aspects of cognition. In the second study, 22 participants (7 TTH, 5 after TTH, and 10 controls) completed paper and pencil mood and cognitive tasks.

**Results:**

In the first study, having a headache was associated with an increase in negative affect both before and after the tasks. Three performance tasks showed impairments when the participants had headaches: logical reasoning was slower and less accurate; retrieval from semantic memory was slower; and reaction times in the categorical search task were slower. Results from the second study confirmed the global increase in negative affect when the person has a TTH. The results confirmed the impairments in the logical reasoning and semantic processing tasks, and also showed that those with a TTH had greater psychomotor slowing and were more easily distracted. Effects did not continue after the headache had gone.

**Conclusion:**

Two small-scale studies have shown that TTH is associated with negative affect and impaired cognitive function. It is now of interest to determine whether OTC treatment can remove these effects.

## Introduction

In general, acute tension-type headaches (TTHs) are temporary and alleviated by rest, relaxation, or over-the-counter medication. However, people with a headache often continue their normal routine, and it is important to understand how such illnesses affect efficiency of mental performance and mood. Research on the effect of acute headaches on mood and cognitive performance is sparse, and the general aim of the present research was to address this gap in our knowledge.

Headache sufferers have been found to report higher levels of chronic negative affect than controls (1, 2). Participants with frequent headaches also score higher on measures of depression than controls (3). A study of 470 headache sufferers revealed them to have significantly higher scores of anxiety and depression using the Hamilton rating scales than healthy controls. Also, 3.4% of these patients achieved the DSM-IV criteria for major depression (4). In addition, the frequency of headache attacks, history of headaches, and gender were correlated within the Hamilton rating scale for both anxiety and depression. Another study concerning the relationship between headaches and mood found that headaches were associated with negative affect, although individual differences existed (5). In addition, mood was found to correlate to headache intensity although this correlation was very small.

The association between headache, usually migraine, and memory impairment has been explored by a number of researchers who have consistently found participants with headaches to show more memory deficits than controls (6, 7). One study (8) assessed the impairments of long- and short-term memory in 26 headache patients and 22 controls with participants completing a test battery, including the Wechsler Memory Scale. Results indicated that those in the headache group had impaired logical and visual memory and worse associative learning compared to normal controls. Few studies have examined effects of acute headache, but there is evidence that confirms that acute headache is associated with deficits in memory recall (9). Only one study has examined the effect of acute headache on attention (10). The results showed that having a headache impaired general performance rather than influencing specific attentional mechanisms.

Other studies have found little evidence for effects of headache on cognition. One study (11) examined the effects of age and being a migraine sufferer on information processing speed and memory in 1,867 people aged between 25 and 80 years and found that suffering from migraine did not influence cognitive performance. On the basis of these findings, it was suggested that migraine does not seriously affect cognitive functioning in adults in the general population. Such a view is supported by more recent research which concluded that the effects of migraine on cognitive neuropsychological functions are trivial to small (12).

Other research has shown that only some individuals are impaired by headache. For example, a population-based study (13) of a random general Danish population revealed 59% of the participants with TTH had moderate or severe impairment of their daily activities because of the headache, yet 41% did not experience impairment in their daily activities. Since, this evidence suggests that acute headaches may cause impairments in the daily activities of some people it has implications for their safe performance when experiencing a headache and reinforces the importance of investigating this area.

Theoretical evidence concerning headaches and how they might affect mood and mental performance is provided by research surrounding neurotransmitters, such as dopamine and serotonin. Clinical evidence has been found to implicate the neurotransmitter dopamine in migraine attacks (14). The research finding suggests that mood changes may thus be related to dopaminergic activation. It has been suggested that the dopaminergic system plays a role in the headache phase by regulating cerebral blood flow or taking part in nociception mechanisms (alert the body to potential damage). Migraine patients, between attacks, have been shown to have a higher responsiveness to acute administration of dopaminergic agents (14). Dopamine is also linked with executive function (components controlled by the frontal lobes) and motor control, and this may help explain why performance in only certain tasks is impaired. It could also be that the susceptibility to headache is caused by serotonin, which is a neurotransmitter implicated to have a central role in depression. Changes in serotonin have been regarded as a possible cause in the development of both migraine and associated depression (15). Therefore, low levels of serotonin may explain the low mood found in people with headaches. Another underlying mechanism is pain. It has been argued (16, 17) that headache-related pain results in an attentional interference effect. However, it would appear that the type of pain interference associated with headache is relatively minor and possibly limited to tasks with a memory component (10).

The aims of the present study were to examine the effects of acute TTH on mood and a range of cognitive functions. Two slightly different sets of task were used to examine the reliability and range of any effects. The research formed part of a larger program of research investigating malaise associated with minor illnesses (18) and methods of removing these behavioral ­problems (19).

## Study 1

This study was carried out with the approval of the local ethical committee (Department of Experimental Psychology, University of Bristol) and the informed consent of the participants. Volunteers were paid for their participation. They were informed that the study was examining the effects of having a headache on mood and cognition.

### Materials and Methods

#### Design

A comparison was made between participants with an acute TTH and those who were healthy. Data were also collected on the second occasion (at least 24 h after the first occasion) when both groups were healthy, and these data were used as covariates to adjust for individual differences. A power calculation showed that the sample size of 12 was selected to be able to detect large effects (Cohen’s *d* > 0.8).

#### Headache

In order to be consistent with previous research on the behavioral effects of having a headache (10), screening was based on the criteria of the Headache Classification Committee of the International Headache Society (20). The headaches studied had to have been present for at least 30 min and no longer than 4 h. Volunteers taking medication for the headache were excluded. The healthy control group was not generally prone to headaches and had not had a headache for at least 7 days. Volunteers with a history of migraine were excluded as were those with any chronic disease or taking medication. Volunteers were given the Psychological Assessment Questionnaire (21), which consisted of five sections covering a description of the headache. In addition, pain was rated before and after testing using a 10-point scale (22). All headaches were located on both sides of the head and were of mild/moderate severity (with a mean pain rating of 4).

#### Participants

The participants were 12 university students. Both headache and control groups had four females and two males. The mean age of the headache group was 25.2 years (SD = 2.4), and the mean age of the control group was 21.0 years (SD = 3.8). Such differences in age have little effect on cognitive performance. Volunteers were paid £10 for their participation.

#### Mood and Performance Tasks

##### Mood

This was measured before and after the cognitive tests using 18 bipolar visual analog scales [e.g., drowsy–alert and tense–calm (23)] presented on the screen of an IBM compatible computer.

##### Performance Tasks

A battery of tests were used, and they were chosen because they have been shown to be sensitive to changes in state and the effects of minor illnesses [e.g., Ref. (24–29)] and measure a range of functions. All of these tests were presented on an IBM compatible PC.

#### Memory Tasks

##### Free Recall Task

In this task (24), a list of 20 words was presented on the PC screen at the rate of one every 2 s. At the end of the list, the volunteer had 2 min to write down (in any order) as many of the words as possible (“immediate recall”).

##### Delayed Recognition Memory Task

This task (24) was at the end of the test session, and volunteers were shown a list of 40 words, which consisted of the 20 words shown at the start of the session plus 20 distracters. They had to decide as quickly as possible whether each word was shown in the original list or not.

##### Logical Reasoning Task

In this task (25, 28), the participants were shown statements about the order of the letters A and B followed by the letters AB or BA (e.g., “A follows B: BA”). They had to read the statement and decide whether the sentence was a true description of the order of the letters. If it was, they pressed the “True” key on the response box; if it was not, they pressed the “False” key. The sentences ranged in syntactic complexity from simple active to passive negative (e.g., “A is not followed by B”). Volunteers carried out the task for 3 min.

##### Semantic Processing Task

This test (25, 29) measures the speed of retrieval of information from general knowledge. Volunteers were shown a sentence and had to decide whether it was true (e.g., “canaries have wings”) or false (e.g., “dogs have wings”). The number completed in 3 min was recorded, as was accuracy.

#### Psychomotor Tasks

##### Simple Reaction Time Task

This simple reaction time task (26) had a variable foreperiod (1–8 s) between the warning signal and the target. A box (the warning signal) was displayed on the screen, and this was followed by a square (the target) being presented in the middle of the box. The participant had to press a key on the response box as soon as the square was detected and following this, another box was presented. This task lasted for 3 min.

##### Five Choice Serial Reaction Time

Five lights were arranged in a pentagon around a central light on the response box. A peripheral light came on and the participant had to press that light, which was followed by the central light becoming illuminated and having to be pressed. When the central light was pressed, the next peripheral button came on and the procedure was repeated. This psychomotor test measures the speed and accuracy of rapid movements in serial response. This task has been widely used to study the effects of stressors and drugs (27).

#### Selective Attention Tasks

##### Focused Attention Task

This choice reaction time task (30) measured various aspects of performance. In this task, target letters appeared as upper case A’s and B’s in the center of the screen. Participants were required to respond as quickly and as accurately as possible to the target letter presented in the center of the screen, ignoring any distracters presented in the periphery. The correct response to A was to press a key with the forefinger of the left hand, whereas the correct response to B was to press a different key with the forefinger of the right hand. Prior to each target presentation, three warning crosses were presented on the screen, in which the outside crosses were separated from the middle one by either 1.02° or 2.60°. The crosses were on the screen for 500 ms and were then replaced by the target letter. The central letter was either accompanied by (1) nothing, (2) asterisks, (3) letters which were the same as the target, or (4) letters which differed from the target. The two distracters presented were always identical, and the targets and accompanying letters were always A or B. Participants were given 10 practice trials followed by 3 blocks of 64 trials. In each block, there were equal numbers of near/far conditions, A or B responses, and equal numbers of the four distracter conditions. The nature of the previous trial was controlled. This test lasted approximately 6 min.

In this task, several aspects of choice responses to a target were measured. The global measures of choice reaction time were mean reaction time and accuracy of response (percent correct) when the target was presented alone or when distracters were present. Long response times (>800 ms) were also recorded.

##### Categorical Search Task

This task (30) was similar to the focused attention task that was previously outlined. Each trial started with the appearance of two crosses either in the central positions occupied by the non-targets in the focused attention task, i.e., 2.04° or 5.20° apart or further apart, located toward either left and right extremes of the screen. The target letter then appeared in place of one of these crosses. However, in this task, participants did not know where the target would appear. On half the trials, the target letter A or B was presented alone, and on the other half, it was accompanied by a distracter, in this task, a digit (1–7). Again the number of near/far stimuli, A versus B responses, and digit/blank conditions were controlled. Half of the trials led to compatible responses (i.e., the letter A on the left side of the screen or letter B on the right), whereas the others were incompatible. The nature of the preceding trial was also controlled. In other respects (practice, number of trials, etc.), the task was identical to the focused attention task. This task also lasted approximately 6 min. As in the focused attention task, several aspects of choice responses to a target are measured. The global measures recorded were choice reaction time and accuracy of response when the target was presented alone in either near or far location. Long response times (>1,000 ms) were also recorded.

##### Sustained Attention Task: Repeated-Digits Vigilance Task

In this task (31), three-digit numbers were shown on the screen at the rate of 100/min. Each was normally different from the preceding one, but occasionally (eight times a minute), the same number was presented on successive trials. Participants had to detect these repetitions and respond as quickly as possible. The number of hits, reaction times for hits, and false alarms were recorded. The task lasted for 3 min.

#### Statistical Analyses

Analyses of covariance were carried out with the control day scores as the covariates and the test day scores as the dependent variables. The headache and healthy groups were the between-subject factor.

### Results

#### Mood

Those with a headache reported a more negative mood both pre- and post-task. This was true for all mood factors (see Table 1). The effects on pre-task alertness and post-task alertness remained significant when a Holm–Bonferroni sequential correction was applied.

**Table 1:** **Effects of headache on mood (scores are the adjusted means and SEs – higher scores = more positive mood)**.

Mood	Healthy	Headache	Statistics
Pre-task alertness	267 (18)	133 (17)	*F* 1,9 = 28.7; *p* = 0.0005
Pre-task hedonic tone	195 (17)	124 (17)	*F* 1,9 = 8.3; *p* = 0.02
Pre-task anxiety	96 (6)	65 (8)	*F* 1,9 = 6.4; *p* = 0.03
Post-task alertness	220 (25)	123 (25)	*F* 1,9 = 7.6; *p* = 0.02
Post-task hedonic tone	167 (18)	101 (18)	*F* 1,9 = 6.6; *p* = 0.03
Post-task anxiety	100 (8)	59 (8)	*F* 1,9 = 12.5; *p* = 0.006

#### Cognitive Performance

**Figure 1:**
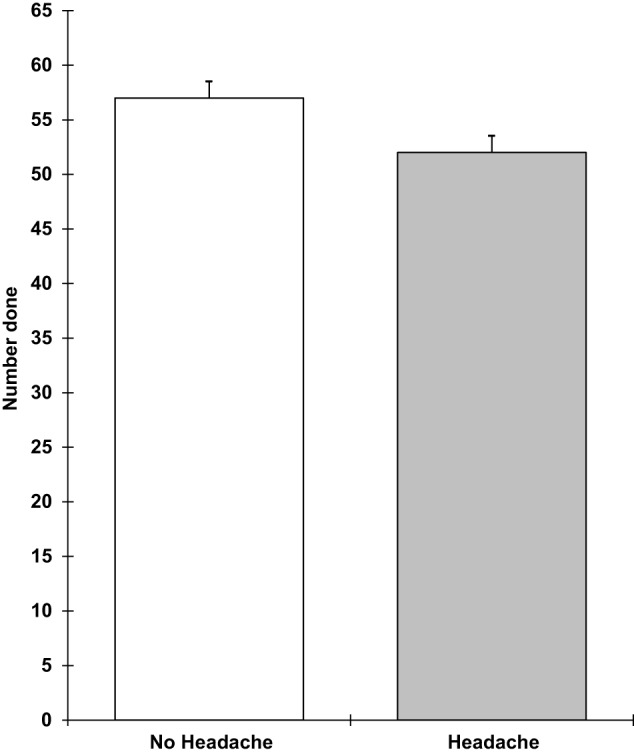
**Verbal reasoning number completed in 3 min (higher scores = faster speed; scores are the adjusted means, SEs shown as bars)**.

**Figure 2:**
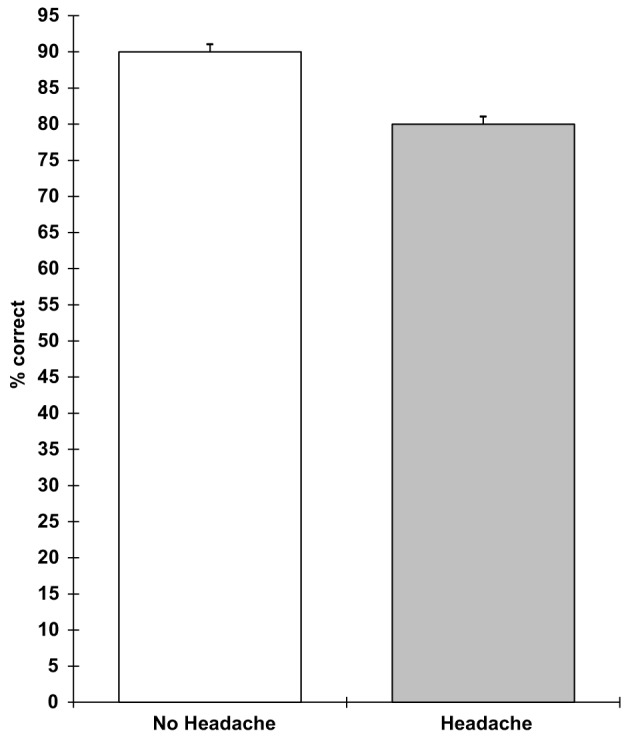
**Verbal reasoning percent correct (higher scores = greater accuracy; scores are the adjusted means, SEs shown as bars)**.

**Figure 3:**
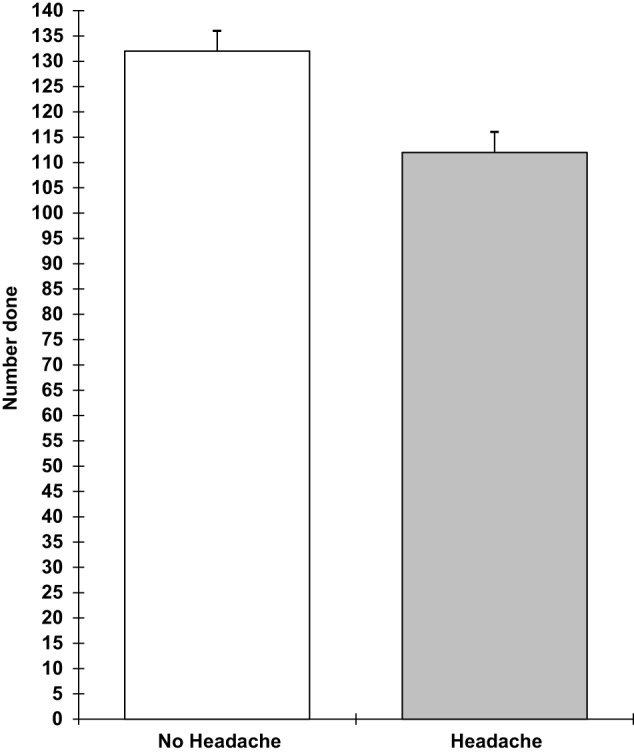
**Semantic processing number completed in 3 min (higher scores = faster speed; scores are the adjusted means, SEs shown as bars)**.

**Figure 4:**
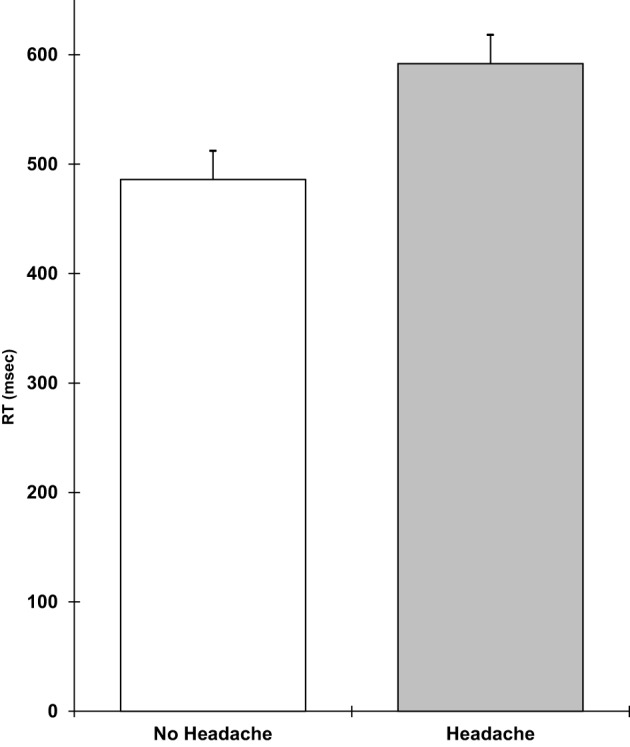
**Categorical search RT in millisecond (higher scores = slower speed; scores are the adjusted means, SEs shown as bars)**.

Analyses of covariance showed that those with a headache were slower on the verbal reasoning task (*F* 1,9 = 5.3; *p* = 0.05), the semantic processing task (*F* 1,9 = 12.1; *p* = 0.007), and the categorical search task (*F* 1,9 = 6.3; *p* = 0.03). They also performed the verbal reasoning task less accurately (*F* 1,9 = 12.5; *p* = 0.006). These results are shown in Figures 1–4. All of the effects remained significant when a Holm–Bonferroni sequential correction was applied. None of the other tasks showed significant effects of having a headache.

### Discussion

The results from the first study suggest that having a headache was associated with a more negative mood. This effect was global in that it was present in all mood dimensions and seen both before and after carrying out the performance tasks. In contrast, the effects of having a headache on the cognitive tasks were selective, with only the verbal reasoning task, semantic processing task, and categorical search task showing significant effects. The next study aimed to replicate these findings and extend them by using a slightly different design and battery of tests.

## Study 2

This study was carried out with the approval of the local ethical committee (School of Psychology, Cardiff University) and the informed consent of the participants. Volunteers were entered into a £50 prize draw on completion of the study.

### Materials and Methods

#### Design

The design of this study was very similar to the previous one except that an “after headache” group was also included. In this condition, participants had reported a headache but been symptom free for at least 2 h when they were tested.

#### Participants

The participants were 22 university students (5 males and 17 females) aged between 18 and 25 years. Participants were recruited by advertisements posted around the campus and from a student participant panel. There were 7 participants in the headache condition, 5 in the “after headache” condition, and 10 healthy controls. These groups did not significantly differ in age.

#### Mood and Performance Tasks

##### Mood

This was measured before and after the cognitive tests using paper and pencil versions of the 18 bipolar visual analog scales used in study 1.

##### Performance Tasks

These were selected to measure a range of mental functions, but the specific tests were largely different from those in the previous study. This meant that one could determine whether effects generalized across functions or were dependent on the specific form of the task.

##### Pegboard Task

This task (32) involved transferring pegs from a full pocket (32 pegs) solitaire set to an empty one. Pegs were transferred one at a time from the full set to the equivalent hole in the empty set. The participants were instructed to use their dominant hand. For right-handed participants, the full set was on the left and the empty set was on the right. The distance between these two pegboards was 21 cm (the width of an A4 page). The first peg to be moved was the one at the extreme top right, and the participant moved down each column ending with the peg in the extreme bottom left of the set. Left-handed participants had the full set on the right and proceeded from left to right rather than from right to left. The time taken to complete this task was recorded.

##### Visual Search Tasks (with and without a Memory Load)

Participants were presented with both simple and complex visual search tasks (32); in the former task, the lines were divided equally into two blocks and the latter task separated equally into four blocks. The task involved detection of one target letter or five target letters, respectively. Each version required searching through 24 lines of 60 single­spaced random capital letters on a page, with the target letter/s presented in the left hand column of sheet. There were between 0 and 3 targets in each line. Participants were instructed to memorize the target/s and then scan the line from left to right, to place a stroke through the targets and not to check back. A different version of this task was used for each test session. The time taken to scan each block (seconds) and the accuracy of target detection were used as measures of performance.

##### Logical Reasoning and Semantic Processing Tasks

Paper and pencil versions of the tasks used in study 1 were in the study 2 test battery.

##### Stroop Color-Word Interference Task

This task (33) measures a person’s ability to resist distraction from irrelevant stimuli. In the simple condition, participants were asked to name the colors of ink sequences on a card. In the more difficult condition, a color name was printed in a different color (e.g., blue), and the participant had to name the color of the ink (red) ignoring the word (blue). The difference between the two conditions is a good measure of susceptibility to distraction from irrelevant cues.

### Results

None of the analyses showed significant differences between the healthy controls and the after-headache group. Planned comparisons between the headache and control groups are, therefore, of main interest.

#### Mood

The results confirmed that those with a headache report a more negative mood and that this was apparent for all measures and before and after the performance tasks (see Table 2).

**Table 2:** **Effects of headache on mood (scores are the adjusted means and SEs – higher scores = more positive mood)**.

Mood	Healthy	Headache	After headache	Statistics
Pre-task alertness	236 (18)	182 (11)	231 (21)	*F* 2,18 = 3.9; *p* = 0.04*
Pre-task hedonic tone	203 (16)	165 (16)	200 (21)	*F* 2,18 = 10.3; *p* = 0.001*
Pre-task anxiety	93 (9)	80 (11)	104 (13)	*F* 2,18 = 3.5; *p* = 0.05*
Post-task alertness	227 (24)	125 (27)	200 (28)	*F* 2,18 = 5.03; *p* = 0.02*
Post-task hedonic tone	188 (13)	144 (15)	209 (17)	*F* 2,19 = 4.3; *p* = 0.03*
Post-task anxiety	81 (8)	64 (9)	86 (11)	*F* 2,18 = 3.6; *p* = 0.05*

*The healthy and headache groups remained significant when Holm–Bonferroni corrections were applied.

#### Performance

**Figure 5:**
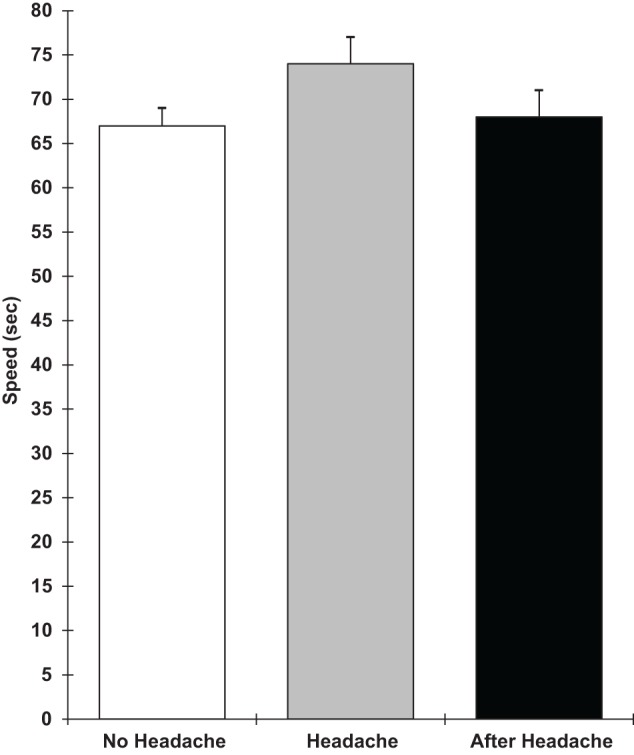
**Pegboard task speed (higher scores = slower speed; scores are the adjusted means, SEs shown as bars)**.

**Figure 6:**
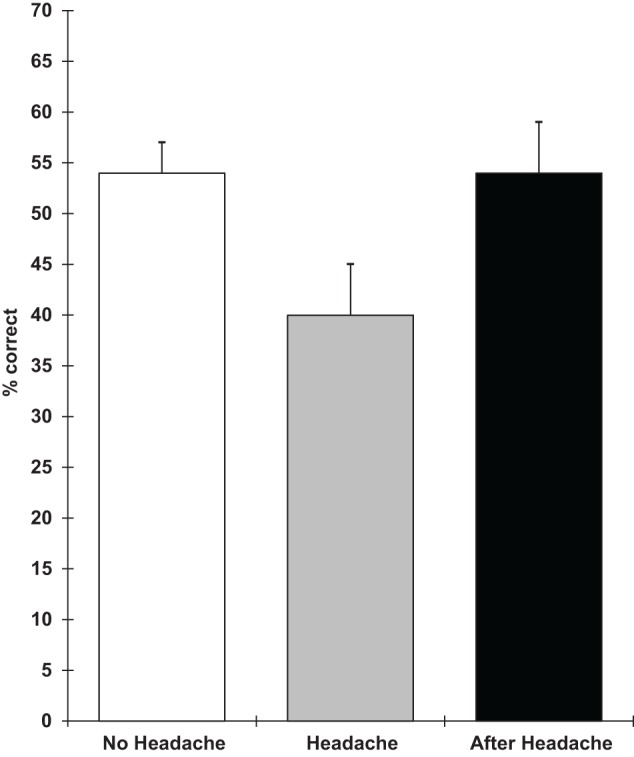
**Memory loaded visual search accuracy (higher scores = greater accuracy; scores are the adjusted means, SEs shown as bars)**.

**Figure 7:**
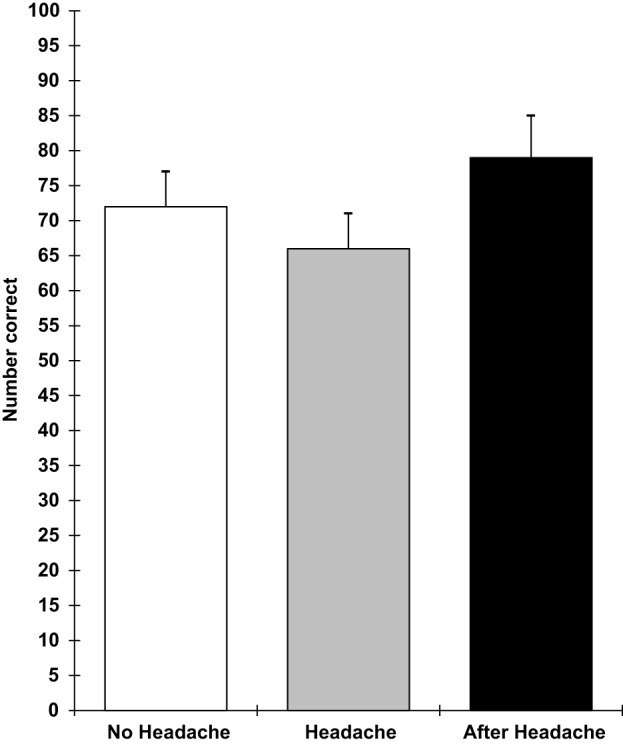
**Semantic memory number correct (higher scores = faster speed; scores are the adjusted means, SEs shown as bars)**.

**Figure 8:**
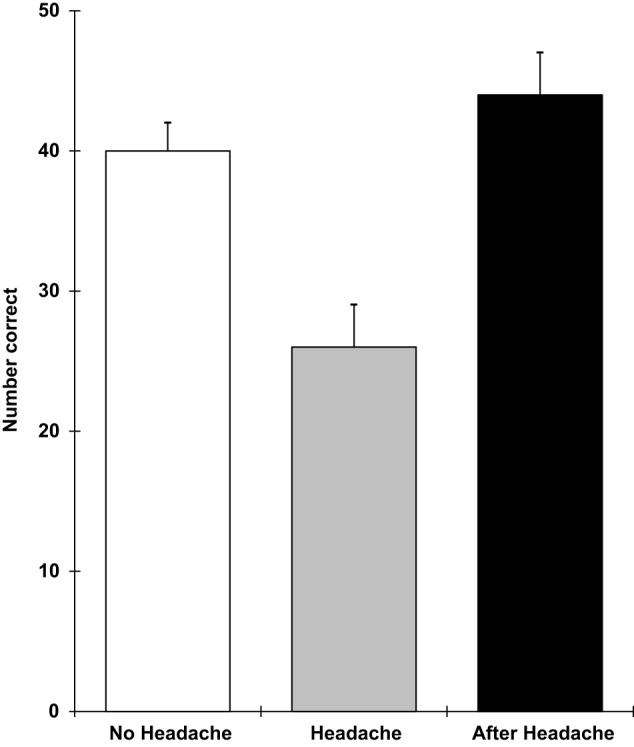
**Logical reasoning number done (higher scores = greater speed; scores are the adjusted means, SEs shown as bars)**.

**Figure 9:**
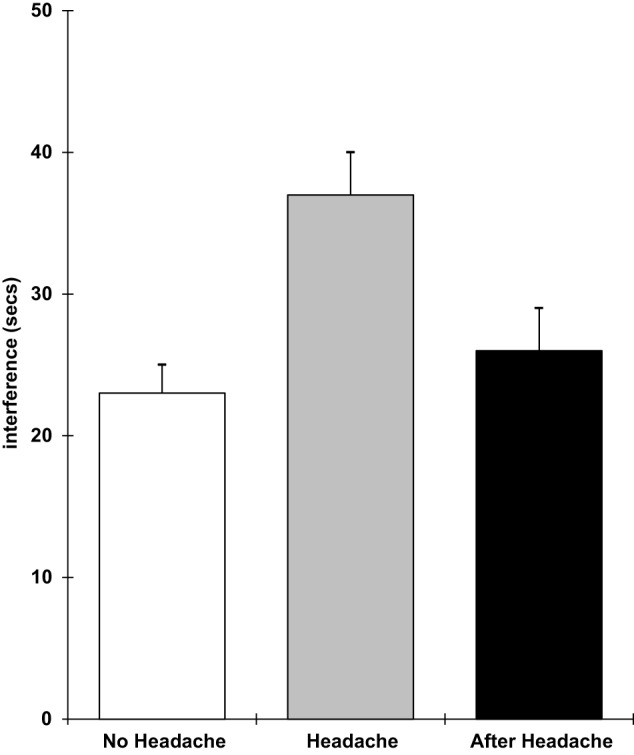
**Stroop interference (higher scores = greater distraction; scores are the adjusted means, SEs shown as bars)**.

The results showed that those with a headache were significantly slower on the pegboard task (*F* 2,18 = 3.78; *p* = 0.05), the verbal reasoning task (*F* 2,18 = 11.9; *p* = 0.0005), and the semantic processing task (*F* 2,18 = 3.5; *p* = 0.05). They also performed the five-item visual search task less accurately (*F* 2,18 = 8.0; *p* = 0.003) and showed greater distraction from irrelevant stimuli in the Stroop task (*F* 2,18 = 8.1; *p* = 0.003). These effects are shown in Figures 5–9. Bonferroni *t*-tests showed that the differences between the healthy and headache groups were significant for all of these variables. These findings show that having a headache has selective effects on cognitive performance with attention tasks (five-item visual search and susceptibility to distraction), working memory (verbal reasoning and five-item search task), retrieval from semantic memory, and motor coordination and movement (the pegboard task) being the functions that were impaired.

### Discussion

The results from the second study confirmed that having an acute TTH was associated with an increase in negative affect, poorer performance on working memory and semantic memory tasks, slower psychomotor performance, and increased distraction from irrelevant stimuli. This suggests that the effects of having a headache are robust and can be observed when different tests are used, and there is variation in background conditions (e.g., duration of test battery and time of day of testing).

Overall, these two small-scale studies show that an acute headache is associated with behavioral changes. It is possible that these changes reflect the demand characteristics of the study (the participants were informed that the research was examining the behavioral effects of having a headache), although such an explanation has difficulty accounting for the selective impairments observed. The increase in negative affect and cognitive impairment clearly are indicators of reduced wellbeing, quality of life, and functionality. As discussed in Section “Introduction,” there are plausible underlying CNS mechanisms that could account for the different effects.

The present research had a number of limitations which largely reflect the fact that these were small-scale preliminary studies of the topic. It is possible that these changes reflect the demand characteristics of the study (the participants were informed that the research was examining the behavioral effects of having a headache), although such an explanation has difficulty accounting for the selective impairments observed. The results could also reflect the nature of the design and a prospective design, with a healthy baseline condition, could eliminate the role of learning effects.

Another major limitation was that the research was not designed to examine the importance of detailed characteristics of the headache symptoms. In addition, it was not designed to identify mechanisms (e.g., localization of the functions affected or neurotransmitter changes) underlying the behavioral effects. This should clearly be an aim for future research. Examination of the functions affected shows that the impairments are not localized. This is true whether one focuses on areas of the brain (e.g., selective attention, logical reasoning, and semantic memory have different neurological bases) or neurotransmitters (e.g., logical reasoning and visual search reflect cholinergic functioning, whereas semantic memory is noradrenergic).

In addition, it is important to determine the real-life impact of such effects on activities involved in work, education, or everyday life (e.g., driving). Furthermore, it is important to conduct trials to examine whether medication will remove the behavioral malaise induced by a headache. Many of the impairments observed in the present study could potentially be removed by compounds such as caffeine. Addition of caffeine to analgesics often leads to an adjuvant effect and may also remove the negative affect and impaired performance (19). There are precedents for following the research strategy outlined here, a good example being psychological studies of upper respiratory tract illnesses (18).

## Author Contributions

Prof. AS designed the study, conducted the analyses, and wrote the paper.

## Conflict of Interest Statement

The author declares that the research was conducted in the absence of any commercial or financial relationships that could be construed as a potential conflict of interest.
